# Cyclin-dependent kinase inhibitors in brain cancer: current state and future directions

**DOI:** 10.20517/cdr.2019.105

**Published:** 2020-03-19

**Authors:** Viktorija Juric, Brona Murphy

**Affiliations:** Department of Physiology and Medical Physics, Royal College of Surgeons in Ireland, Dublin D02, Ireland.

**Keywords:** Cyclin-dependent kinases, cyclin-dependent kinase inhibitors, gliomas, glioblastoma, clinical trials, resistance

## Abstract

Cyclin-dependent kinases (CDKs) are important regulatory enzymes in the normal physiological processes that drive cell-cycle transitions and regulate transcription. Virtually all cancers harbour genomic alterations that lead to the constitutive activation of CDKs, resulting in the proliferation of cancer cells. CDK inhibitors (CKIs) are currently in clinical use for the treatment of breast cancer, combined with endocrine therapy. In this review, we describe the potential of CKIs for the treatment of cancer with specific focus on glioblastoma (GBM), the most common and aggressive primary brain tumour in adults. Despite intense effort to combat GBM with surgery, radiation and temozolomide chemotherapy, the median survival for patients is 15 months and the majority of patients experience disease recurrence within 6-8 months of treatment onset. Novel therapeutic approaches are urgently needed for both newly diagnosed and recurrent GBM patients. In this review, we summarise the current preclinical and clinical findings emphasising that CKIs could represent an exciting novel approach for GBM treatment.

## Introduction

Cyclin-dependent kinases (CDKs) are a family of enzymes-serine threonine kinases - that, under normal physiological conditions, play significant roles in controlling cell-cycle progression and transcription regulation^[1]^. Overexpression of some CDKs and their associated cyclins, as well as downregulation of CDK inhibitors (CKIs), can lead to abnormal cellular proliferation and cancer progression^[2]^. Due to such dysregulation of CDKs in many cancers, targeting of this family of enzymes has emerged as a promising strategy in the treatment of multiple cancer types, including blood and solid tumours^[3,4]^. Targeting the cell cycle via the CDK4/6-Rb axis has proven the most successful approach in the clinic to date. The FDA has approved the CDK4/6 inhibitors palbociclib, abemaciclib and ribociclib as treatments for hormone receptor-positive (HR+) metastatic breast cancer (mBC), in combination with endocrine therapy^[5-7]^.

Glioblastoma (GBM) tumours also harbour genomic alterations that lead to the constitutive activation of CDKs, resulting in tumour proliferation^[8]^. Hence, the potential of CKIs as a novel treatment option for GBM patients has been investigated. Preclinical studies in cell lines and animal models have yielded positive results and provided hope for the future utilisation of these inhibitors as a novel treatment option for GBM patients^[9,10]^. However, clinical trials undertaken to test CKIs in glioma patients have not proven successful^[11]^. In this review, we discuss the main findings from the application of CKIs as a potential treatment for glioma, in both preclinical and clinical studies. We highlight the main drawbacks and examine the future opportunities in developing these drugs as single or combinational treatment options for glioma patients with the specific focus on GBM.

## GBM: standard of care and treatment resistance

Gliomas represent the most common primary malignancy in the central nervous system (CNS) including astrocytomas, oligodendrogliomas and ependymomas and account for approximately 80% of malignant brain and CNS tumours^[12]^. Based on clinical and biological characteristics, gliomas can be subdivided into two main categories, low-grade glioma and high-grade glioma. Based on their histological appearance, the World Health Organisation (WHO) categorises gliomas using a classification index, ranging from I to IV, which grades gliomas according to disease prognosis^[13]^. WHO grades I and II have been referred to as low-grade gliomas, with a possibility of cure following surgical resection alone, while more rapidly progressing tumours are referred to as high-grade gliomas. Grade III neoplasms are associated with histological signs of malignancy, nuclear atypia, mitotic activity and high proliferative lesions. GBM is the highest-grade glioma tumour (Grade IV) and the most malignant form of astrocytic glioma with extremely poor treatment response and prognosis^[13,14]^. Two types of GBM usually occur: primary and secondary. Primary GBM develops *de novo* from glial cells or supportive tissues of the brain, accounts for approximately 90% of GBM cases and is more common in older patients. Secondary GBMs develop from pre-existing low-grade gliomas, accounting for 10% of GBM cases and are more common in younger patients^[15]^. Some DNA alterations are shared between primary and secondary GBM, including *TP53* mutations and *EGFR* overexpression^[16]^. On the other hand, loss of *PTEN* has been typically observed only in primary GBM, whereas secondary GBM often contains loss of chromosome 19q along with *TP53*^[17,18]^. As for the median survival rate, primary GBM shows worse prognosis when compared to secondary GBM following maximal therapy, with overall survival of 15 months and 31 months, respectively^[19]^. Using genomic profiling to classify GBM tumours, Verhaak *et al*.^[20]^ divided GBM into four subtypes. Proneural, mesenchymal, classical and neural subtypes were distinguished upon analysis of an 840-gene signature^[20]^. Proneural subtype is described as being less aggressive with some similarities to secondary GBM, including mutations of *TP53* contributing to the genetic changes that lead to tumour progression^[21-23]^. Additionally, alterations of *PDGFRA* and point mutations in *IDH1* are found in the proneural subtype^[20]^. On the other hand, mesenchymal, classical and neural subtypes are more aggressive with high expression of genes responsible for cell proliferation, angiogenesis and invasion^[24]^. Classical subtype is characterised by high levels of *EGFR* amplification which is not commonly found in other subtypes. *CDKN2A* homozygous deletion is also commonly found in this subtype^[20]^. Interestingly, a lack of *TP53* mutations is observed in the classical subtype even though it is the most common mutation in GBM^[25]^. Mesenchymal subtype is characterised by a high frequency of *NF1* abnormalities and markers associated with inflammation, wound healing, and NF-κB signalling pathways^[20,24]^. Neural subtype is associated with *EGFR* overexpression and has a strong enrichment for genes differentially expressed by neurons^[20]^. More recently, the neural subtype has been discarded as it showed no tumour specificity in a gene set enrichment analysis using a 50-gene signature per subtype^[26]^. Other types of classification are known and, while such subtyping of GBM tumours will hopefully lead to more targeted therapies in the future, such classifications are not currently applied in the clinic.

The standard treatment for newly diagnosed GBM patients consists of maximal neurosurgical resection with the overall aim to remove all visible tumour such that no tumour is observed post-surgery upon magnetic resonance imaging. Following surgery, adjuvant treatment is advocated and the so-called Stupp protocol is widely used^[27]^. According to this protocol, patients should receive concomitant radiotherapy (RT) and temozolomide (TMZ) followed by adjuvant TMZ. Radiotherapy consists of fractionated focal irradiation at a dose of 2 Gray (Gy) per fraction given once daily, five days per week over a period of six weeks, for a total dose of 60 Gy. Concomitant chemotherapy consists of TMZ at a dose of 75 mg/m^2^ per day, given seven days per week during radiotherapy. Upon completion of chemo- and radiotherapy, there should be a 4-week break. After that, patients should receive up to 6 cycles of adjuvant TMZ, given for five days every 28 days, with a starting dose of 150 mg/m^2^ for the first cycle. This dose is subsequently increased to 200 mg/m^2^ at the beginning of the second cycle, as long as there are no hematologic toxic effects^[28]^. To date, the addition of TMZ to the armamentarium remains the biggest significant advance in the management of GBM. When successful, TMZ induces cell death by causing DNA double strand breaks that eventually lead to growth arrest and activation of cellular apoptosis^[29,30]^. Unfortunately, however, its benefit is limited to prolonging patient survival rather than being a curative treatment adjunct. Most patients experience tumour relapse within seven months of treatment onset, while a large proportion gain no survival advantage to TMZ therapy at all^[31]^. Several factors contribute to this poor survival, including but not limited to, patient condition, tumour location, and the heterogeneous instability within GBM cells^[32]^. Another potential mechanism of treatment failure is the blood-brain barrier (BBB), which consists of specialised endothelial cells with tight junctions and transport proteins that serves to restrict brain uptake of drugs, including systemic chemotherapies^[33]^. Another significant factor in such disappointing patient survival rates is TMZ resistance. TMZ, as well as most other anti-cancer therapies, exerts its cytotoxic effect by triggering apoptosis in cancer cells^[30,34]^, thus defective apoptosis provides a significant mechanism by which TMZ treatment fails in patients. A key marker of GBM responsiveness to TMZ is the methylation status of the O6-methylguanine-DNA methyltransferase (MGMT)^[27]^. MGMT mediates the direct removal of O6-methylguanine lesion, the most common cytotoxic lesion induced by TMZ. Approximately 50% of GBM patients do not respond to TMZ treatment, most likely due to MGMT overexpression, while epigenetic silencing of MGMT via methylation of the MGMT gene promoter results in increased genome instability and chemosensitivity to TMZ^[35]^. Overexpression of numerous survival proteins and attenuated levels of several proapoptotic proteins also contribute to the apoptosis resistance in GBM^[36]^; Bcl-2, Bcl-xL, Bax and myeloid cell leukemia 1 (Mcl-1) proteins are among most studied so far^[37]^.

Undoubtedly, new treatment options are needed. Maximising the apoptotic activity within GBM cells, by using other death-inducing stimuli, may help to reduce the resistance of GBM to cell death-inducing treatment strategies^[38]^. Recent studies showed that the treatment resistance evident in GBM can be overcome by using drugs targeting not only the expression of anti-apoptotic proteins but also those that target cell-cycle dysregulation^[39]^, including but not limited to, CDK4/6/cyclin D overexpression. These attributes are evident in CKIs.

## CDKs and their physiological role

**Figure 1:**
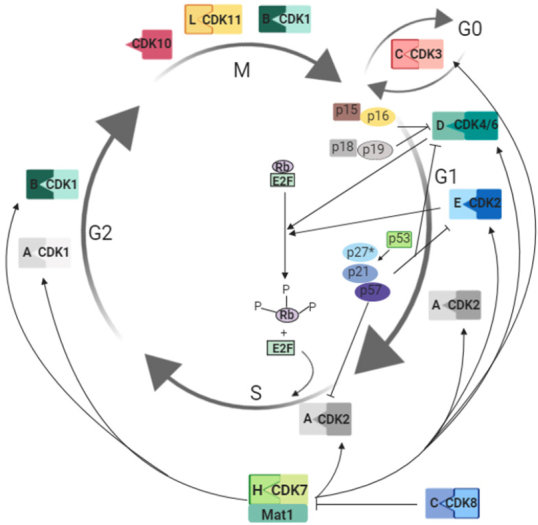
CDK-cyclin complexes and their function in cell cycle. Each CDK is shown in complex with its corresponding cyclin. For clarity, only few substrates are included. CDKs 1, 2, 4 and 6 are classical CDKs involved in the regulation of cell-cycle progression and transition from G1 to S phase. CDK1 in complex with cyclins A and B regulate G2/M transition and mitosis together with CDK11-CycL. CDK3 is involved in the control of the cell-cycle interphase. Activity of these CDK-cyclin complexes is regulated by other CDKs including CDK7 (in complex with its cyclin H and Mat1), CKIs (p15, p16, p18, p19, p27, p21 and p57) and regulatory elements such as the Rb. CDK7 functions in the regulation of several other CDKs via phosphorylation and is controlled by CDK8 in complex with its cyclin C. *Nonphosphorylated p27. Single letters stand for cyclins, for instance H is cyclin H. CKIs: cyclin-dependent kinase inhibitors; CDK: cyclin-dependent kinase; Rb: retinoblastoma protein; G1: growth phase; S: DNA synthesis; G2: second growth phase; M: mitosis; G0: resting phase; E2F: E2 transcription factor

The mammalian CDK family has more than 20 known members^[3]^. The first CDK, now known as CDK1, was discovered in yeast where it was shown to be essential for progressing the cell cycle^[40]^. Soon after homologues of CDK1 were identified in human cells^[41]^. Unsurprisingly, the cell-cycle process is strictly controlled to ensure successful cell division. Cyclin-dependent kinases tightly regulate progression through the G1 (growth phase), S (DNA synthesis), G2 (second growth phase) and M (mitosis) phases of the cell cycle, working in conjunction with their associated cyclins^[42,43]^. CDKs 1-4, 6 and 11 control varying aspects of cell division including DNA replication, mitotic progression, and response to regulatory growth signals^[44-50]^. In association with their cyclins, CDKs 2, 4 and 6 regulate progression from G1 to S phase. CDK3 is involved in G0 and interphase and not much is known about it since it is inactive in most strains of laboratory mice^[51]^. In conjunction with cyclins E and A, CDK2 is involved in regulating progression from S to G2 phase. CDK1 with cyclins A and B is involved in regulating the G2 phase. Finally, mitosis is under the control of CDK1-CycB and CDK11-CycL complexes [Table 1 and Figure 1].

**Table 1:** Most extensively studied CDKs and their known physiological roles

CDKs	Cyclin partner(s)	Cellular functions	Ref.
CDK1	Cyclin A, B1	DNA structure checkpoints during late G2 and the spindle assembly checkpoint during mitosis	[44,45]
CDK2	Cyclin A	Control of G1-S phase of cell cycle (DNA replication)	[44,45]
	Cyclin E1, E2	Rb/E2F transcription	[44,45]
CDK3	Cyclin C	Control of interphase NHEJ-mediated DNA damage repair	[46]
CDK4	Cyclin D	Control of G1 phase of cell cycle Rb/E2F transcription	[47]
CDK5	p35, p39, Cyclin I	Senescence, post-mitotic neurons	[52-54]
CDK6	Cyclin D	Control of G1 phase of cell cycle Rb/E2F transcription	[50]
CDK7	Cyclin H	CAK RNAP II transcription (initiation to elongation)	[55-57]
CDK8	Cyclin C	RNAP II transcription (transcriptional repressor)	[58,59]
CDK9	Cyclin T1, T2a, T2b	RNAP II transcription	[60,61]
	Cyclin K	DNA damage response	[62]
CDK10	Cyclin T	G2/M transition, suppression of Ets2 transactivation domain	[61,63]
CDK11	Cyclin L	G2/M transition, RNA processing	[48,49]

G1: growth phase; S: DNA synthesis; G2: second growth phase; M: mitosis; CDKs: cyclin-dependent kinases; Rb: retinoblastoma protein; NHEJ: non-homologous end joining; RNAP II: RNA polymerase II; CAK: cdk-activating kinase; E2F: E2 transcription factor; Ets2: E26 transformation-specific transcription factor 2

The kinase activity of CDK-cyclin complexes is also highly controlled by an abundance of CKIs, which serve as brakes to control cell-cycle progression according to the conditions in cells^[64]^. CKIs are divided into two groups. The Ink4 family members, including p16^INK4a^, p15^INK4b^, p18^INK4c^ and p19^INK4d^, are mainly involved in the regulation of CDKs 4 and 6^[65]^. On the other hand, the Cip/Kip family members, namely p21^Cip1^, p27^Kip1^ and p57^Kip2^, regulate the activities of cyclin D-, E-, A- and B-dependent kinase complexes, mostly CDKs 2, 4 and 6^[66-68]^ [Figure 1]. Moreover, an important substrate for CDKs is the retinoblastoma protein (Rb)^[69,70]^. During cell division, Rb binds to the transcription factor E2 transcription factor (E2F) and inhibits the activity of the E2F complex, thus preventing cell-cycle progression from the G1 phase to the S phase^[71]^. Phosphorylation of Rb is primarily initiated by the CDK4/CDK6-CycD complex, followed by additional phosphorylation by the CDK2-CycE complex^[72]^, resulting in the inactivation of Rb and cell-cycle progression^[69,73,74]^ [Figure 1].

**Figure 2:**
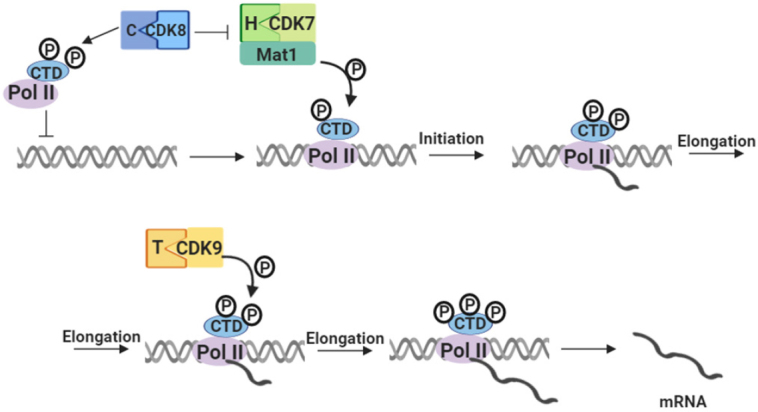
CDKs and their function in RNA PolII transcription. CDKs 7 and 9 are involved in RNA polymerase II activity by directly phosphorylating CTD and thus control the generation of mRNA transcripts. CDK7-CycH-Mat1 complex is under control of CDK8-CycC complex, which acts as its inhibitor. CDK8-CycC complex also prevents binding of RNA PolII to promoter DNA region by its phosphorylation. CDK7-CycH-Mat1 complex phosphorylates RNA PolII CTD domain and enables transcription initiation. Further phosphorylation with CDK9-CycT leads to transcription elongation and mRNA production. Single letters stand for cyclins, for instance T is cyclin T. Pol II: RNA polymerase II; P: phosphatidyl group; CDK: cyclin-dependent kinase; CTD: carboxy-terminal domain; mRNA: messenger RNA

Besides their well-established function in the cell cycle, it is now clear that CDKs, cyclins and CKIs play crucial roles in other cellular processes such as transcription, mRNA processing, epigenetic regulation, metabolism, stem cell self-renewal and differentiation of nerve cells^[75]^. For instance, CDKs 8 and 9 are primarily implicated in transcriptional regulation^[75-77]^ [Table 1 and Figure 2]. Phosphorylation of a key threonine residue located within the activating segment, also known as T-loop, of the CDK subunit is required for full kinase activity^[55]^. This step is carried out by the CDK-activating kinase, known as CDK7, which becomes activated by binding to cyclin H. CDK9, together with cyclin T1, comprises a positive transcription elongation factor b, which plays a key role in the regulation of RNA polymerase II (RNAP II)-mediated transcription via phosphorylation of RNAP II^[60]^. This phosphorylation releases RNAP II from its paused state, triggering transcriptional elongation and ultimately mRNA transcript formation. CDK5 is mostly active in post-mitotic neurons and is essential for neuronal cell-cycle arrest and differentiation^[52]^. In addition, some studies have shown dysregulation of CDK5 in neuronal diseases such as Alzheimer’s disease, Parkinson’s disease and Huntington’s disease, leading to neurotoxicity^[52]^. In contrast to other CDKs, CDK5 is activated by binding to p35 and p39, which have structural homologies to typical cyclins^[53]^ but can also be activated by cyclins I^[54]^. Inhibition of transcriptional CDKs primarily affects the accumulation of transcripts with short half-lives, including anti-apoptotic family members, Mcl-1 and X-linked inhibitor of apoptosis protein (XIAP)^[10,78]^.

## CKIs as approved targeted therapy in cancer treatment

The essential roles of CDKs in the intracellular control of the cell cycle and regulation of transcription and DNA repair^[79]^ make them highly suitable as targets of inhibitors for the treatment of cancer.

Separate Phase III clinical trials using CDK4/6 inhibitors, palbociclib, abemaciclib and ribociclib showed an increase in median progression-free survival (PFS) of patients, approximately 7-10 months, when compared to placebo treated groups^[5-7]^. These highly selective CDK4/6 inhibitors are currently used clinically for the treatment of HR+ mBC patients and were the first CKIs to receive FDA approval for cancer patients^[5-7]^.

After Finn *et al*.^[5]^ demonstrated synergy between palbociclib and endocrine therapy in luminal oestrogen receptor-positive human breast cancer cell lines, it was first tested in Phase I clinical trials in patients with other solid tumours and non-Hodgkin’s lymphoma^[80]^. Palbociclib was well tolerated by patients with advanced solid tumours, with the dose-limiting toxicities mainly related to myelosuppression^[80]^. Palbociclib was next studied in a randomised Phase II clinical trial in metastatic hormone receptor positive breast cancer, in which the combination of palbociclib and endocrine therapy significantly prolonged progression-free survival of patients over endocrine therapy alone^[5]^. Recently, palbociclib has been shown to be effective when combined with fulvestrant in patients with HR+ and human epidermal growth factor receptor 2 negative (HER2-) advanced breast cancer who have stopped responding to endocrine therapy^[81]^. Phase III clinical trials using palbociclib in combination with non-steroid aromatase inhibitor in postmenopausal women with HR+ mBC showed median PFS of 24.8 months compared to 14.5 for placebo treated group^[82]^. Another trial using palbociclib in combination with fulvestrant showed benefits for patients with HR+ mBC that relapsed or progressed during endocrine therapy^[83]^.

Ribociclib is another highly selective CDK4/6 inhibitor approved for the treatment of HR+ mBC patients. A recent Phase III clinical trial using ribociclib in combination with letrozole in HR+ mBC showed an increase in PFS in patients: 25 months *vs*. 16 months compared to placebo group^[6]^. MONALEESA-3 study showed an eight-month improvement in PFS when ribociclib was used in combination with fulvestrant compared to fulvestrant alone^[84]^. Another significant trial including ribociclib, MONALEESA-7, demonstrated improved PFS from 13 to 23.8 months when ribociclib was administrated with tamoxifen/goserelin or non-steroidal aromatase inhibitor/goserelin compared to placebo^[85]^.

The third approved CKI for HR+ mBC treatment is abemaciclib. Low IC_50_ values for CDKs 4 and 6, ranging from 2 to 10 nM, respectively, in addition to CDK 9 inhibition make this CKI most potent in HR+ mBC treatment^[86]^. In combination with either letrozole or anastrozole, abemaciclib significantly improved PFS to 14.7 months^[7]^. When combined with the oestrogen receptor degrader fulvestrant in the MONARCH 2 study, abemaciclib led to PFS improvement, from 9.3 to 16.4 months^[87]^.

## CKIs in glioma treatment - preclinical studies

**Figure 3:**
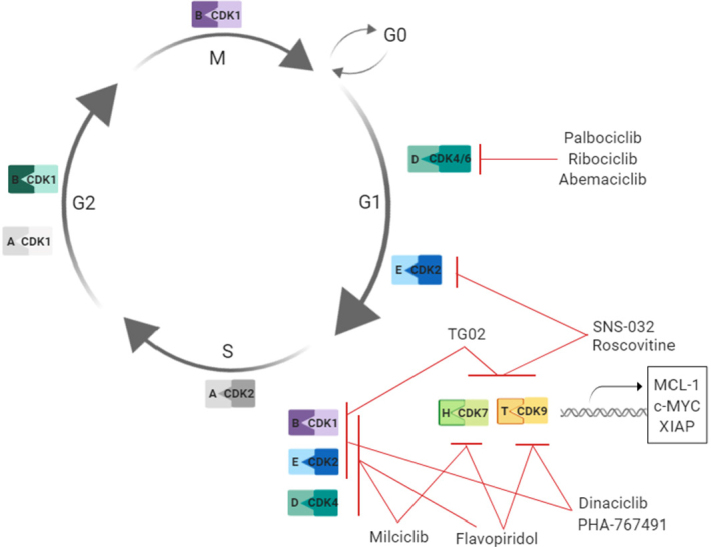
Cell-cycle regulation, CKIs studied in glioma treatment and their targets. Cell-cycle progression in cancer and the role of cyclin-dependent kinases inhibitors used in glioma studies. CDK2-cyclin E complex phosphorylates Rb leading to loss of repression of E2F factors, resulting in cell-cycle progression. Blocking the CDK2 with CKIs leads to cell-cycle arrest by preventing cell-cycle progression from G1 to S phase. Phosphorylation of carboxy-terminal domain of RNAP II by CDK9-cyclin T and CDK7-cyclin H complexes leads to transcription elongation. Blocking of CDKs 9 and 7 leads to transcription inhibition and downregulation of short-lived mRNAs, including MCL-1, c-MYC and XIAP. Single letters stand for cyclins, for instance E is cyclin E. G1: growth phase; S: DNA synthesis; G2: second growth phase; M: mitosis; G0: resting phase; CDK: cyclin-dependent kinase; MCL-1: myeloid leukaemia 1 protein; c-MYC: avian myelocytomatosis virus oncogene cellular homolog; RNAP II: RNA polymerase II; Rb: retinoblastoma protein; XIAP: X-linked inhibior of apoptosis protein; E2F: E2 transcription factor

Flavopiridol is one of the most extensively investigated CKIs in the treatment of glioma. Flavopiridol directly inhibits CDKs 1, 2 and 4^[88,89]^ and induces cell-cycle arrest in G1 or G2 phase [Figure 3]. Additionally, it inhibits CDK7-CycH, thus preventing the phosphorylation and subsequent activation of several CDKs involved in the regulation of cell-cycle progression^[90]^. Furthermore, flavopiridol down-regulates cyclin D1, the cyclin associated with CDKs 4 and 6^[91]^. Promising results were obtained in glioma cell lines when flavopiridol was administered as a single agent^[92]^. In this study, the authors showed that flavopiridol induced caspase-independent cell death in a panel of GBM cell lines independently of p53 and Rb status^[92]^. Newcomb *et al*.^[93]^ further showed that flavopiridol inhibited the growth of GL261 gliomas in subcutaneous and intracranial models *in vivo*. Another study showed enhanced cytotoxicity when flavopiridol was combined with TMZ, in both glioma cells *in vitro* and in nude mice with xenografted U87MG cells^[9]^. However, this first-generation pan-CDK inhibitor failed to enter clinical trials as its low specificity for CDKs resulted in a significant toxicity profile.

Roscovitine, another pan-CDK inhibitor, was first described by Meijer *et al*.^[94]^. They demonstrated the drug’s ability to inhibit CDKs 1, 2 and 5 [Figure 3]. More recent research has revealed that roscovitine also inhibits CDKs which are not directly involved in the regulation of the cell cycle, namely CDKs 7 and 9^[95,96]^. Kolodziej *et al*.^[96]^ showed anti-tumour effects of roscovitine when administrated as a single agent in the GBM cell lines A172 and G28. Several groups have shown that roscovitine can induce significant levels of apoptosis in GBM cell lines upon co-treatment with the death receptor ligand TNF-related apoptosis-inducing ligand (TRAIL)^[97,98]^. Resistance to TRAIL-mediated apoptosis in GBM was overcome by roscovitine’s ability to downregulate survivin, XIAP and Mcl-1^[97,98]^. More recently, using glioma cell lines and an *in vivo* orthotopic glioma model, the combination of roscovitine with TMZ was shown to result in increased autophagy and caspase-3 mediated cell death^[99]^. Menn *et al*.^[100]^ showed the ability of roscovitine to cross the BBB in healthy adult rats while studying ischemic stroke. While roscovitine has not been tested in GBM patients, it is currently in clinical trials for non-small cell lung carcinoma (NCT00372073), rheumatoid arthritis (ISRCTN36667085) and Cushing’s disease (NCT03774446).

Milciclib is another CKI developed to target CDKs 2, 7, 4, 5 and 1 [Figure 3]. This CKI was first tested in human ovarian carcinoma cells. Upon demonstration of *in vitro* effectiveness^[101]^, it entered Phase I and II studies in adult patients with advanced/metastatic solid tumours (NCT01300468). It has been shown that milciclib induces autophagic cell death in a panel of GBM cell lines^[102]^. In this same study, it was shown that milciclib effectively crossed the BBB and reduced tumour size in two *in vivo* GBM models^[102]^. Milciclib has also shown anti-tumour activity when used in combination with TMZ and RT in a xenograft model of GBM^[102]^. Overall, these results highlight the potential for future investigations on the applicability of milciclib for the treatment of GBM patients.

Dinaciclib is another inhibitor that targets multiple CDKs^[103]^. Dinaciclib inhibits CDKs 1, 2, 5 and 9 [Figure 3], thus inhibiting CDKs involved in both cell-cycle regulation and transcription^[104,105]^. *In vitro* studies by Jane and colleagues showed that dinaciclib induced cell-cycle arrest in glioma cells regardless of p53 mutational status^[106]^. Although antiproliferative effects were present, there was no evidence of cell death when dinaciclib was used as a single agent^[106]^. In combination with the Bcl-2 and Bcl-xL inhibitor ABT-737, dinaciclib induced apoptotic cell death in glioma cells, while further mechanistic investigations showed that the observed cell death was a consequence of Mcl-1 downregulation^[106]^. Dinaciclib has a lower toxicity profile compared to other pan-CKIs but the exact reasons behind this are still unknown. It could be due to more specific targeting of CDKs compared to other pan-CKIs or due to having fewer non-CDK off-target effects^[107]^. As a result of lower toxicity profile, dinaciclib has entered clinical trials and is currently in Phase III trial for refractory chronic lymphocytic leukaemia (NCT01580228) and the results from this trial are eagerly anticipated.

SNS-032 is a potent CKI that inhibits both the cell cycle via CDKs 2 and 7 and transcription via CDKs 7 and 9 [Figure 3]. SNS-032 was shown to be effective in GBM cell lines, where it inhibited cellular proliferation in a dose-dependent manner^[108]^, by blocking the production of vascular endothelial growth factor. Ali *et al*.^[109]^ additionally showed that SNS-032 prevented hypoxia-mediated U87MG cell invasion, via its ability to interfere with the expression of HIF-1α and its trans-regulating factors. SNS-032 was also tested in lymphocytic leukaemia where Chen *et al*.^[110]^, showed the ability of SNS-032 to downregulate the anti-apoptotic protein Mcl-1 and induce apoptotic cell death, to a much higher extent when compared to flavopiridol and roscovitine. These results led to a clinical study using SNS-032 in chronic lymphocytic leukaemia and multiple myeloma (NCT00446342)^[111]^. The results from this clinical study remain unpublished.

Erbayraktar *et al*.^[112]^ used the cell division cycle 7-related protein kinase (Cdc7) inhibitor PHA-767491 to enhance replicative stress in glioblastoma cells. They showed that Cdc7 inhibition resulted in inhibition of cellular proliferation, apoptotic cell death induction and blocked GBM invasiveness. PHA-767491 is a first-generation Cdc7 inhibitor with well described anti-tumour activity and more improvement in this field is expected.

Table 2 summarises CKIs used in glioma preclinical studies either as a single treatment or in combination with TMZ/other drugs.

**Table 2:** CKIs used in glioma preclinical studies

CKI drugs	Targets	Combination with other drugs	BBB penetration	Ref.
Flavopiridol (alvocidib)	CDK 1, 2, 4, 9, 7	Not reported	Yes	[92]
		Not reported		[113]
		Not reported		[93]
		Temozolomide		[9]
		Not reported		[91]
Roscovitine (Seliciclib, CYC202)	CDK 2, 5, 7, 9	TRAIL	Yes	[97]
		TRAIL		[98]
		Not reported		[96]
		Temozolomide		[99]
Milciclib (PHA-848125)	CDK 2, 4, 7, 1	Temozolomide	Yes	[102]
Dinaciclib (SCH727965, MK-7965)	CDK 2, 5, 1, 9	ABT-737	Not reported	[106]
SNS-032 (BMS-387032)	CDK 2, 7, 9	Not reported	Not reported	[108]
		Celecoxib, SU 5416 and GM 6001		[109]
PHA-767491	Cdc7; CDK 9, 2, 1	Not reported	Not reported	[112]

CKIs: cyclin-dependent kinase inhibitors; CDK: cyclin-dependent kinase; BBB: blood-brain barrier; TRAIL: TNF-related apoptosis-inducing ligand; Cdc7: cell division cycle 7-related protein kinase

## CKIs reached clinical studies in glioma

After being extensively investigated in different types of cancer and clinically approved for patients with ER+ mBC, palbociclib entered studies for glioma treatment. Initial results showed promise, with palbociclib being able to penetrate the BBB and reduce tumour growth in glioblastoma intracranial xenografts^[114]^. Palbociclib was also shown to be effective *in vitro* and *in vivo* as a single treatment and in combination with GBM standard of care, radiotherapy and TMZ^[114]^. A recent publication showed the potential of palbociclib combined with RT in patient-derived glioblastoma cell lines^[115]^. Despite these promising results obtained in preclinical studies, palbociclib was inefficient when tested as a monotherapy in a Phase II clinical trial for recurrent GBM patients with detectable Rb expression (NCT01227434)^[11]^. More recently, a clinical trial examining the effectiveness of palbociclib in anaplastic oligodendrogliomas was stopped early due to lack of efficacy, with 74% of evaluable patients progressing within six months, despite good drug tolerance and exposure^[116]^. Among the many on-going or terminated clinical trials using palbociclib in glioma treatment, these are the only studies with published results while findings from other clinical studies are highly anticipated.

Ribociclib, an orally bioavailable CDK4/6 inhibitor [Figure 3], is currently under investigation for the treatment of paediatric CNS tumours^[117]^. Initially tested was ribociclib’s ability to reach its targets in the brain. This characteristic was tested in both non-tumour bearing mice and in mice bearing DIPGx7 (glioma) cortical allograft tumours^[117]^. Ribociclib was shown to have adequate CNS exposure and will be further investigated as a treatment option for childhood brain tumours. However, a recent study showed that the brain penetrance of ribociclib *in vivo* is restricted by the ABCB1 transporter^[118]^, leading to the suggestion that co-administration of the ABCB1 inhibitor elacridar could dramatically improve the brain penetrance of ribociclib^[118]^ and further investigations are underway. A clinical trial testing ribociclib in recurrent GBM patients showed that ribociclib can cross the tumour-brain barrier at pharmacologically-active concentrations and suppress tumour proliferation. This Phase II study suggested however that ribociclib is ineffective as a monotherapy, and proposed that the addition of an mTOR inhibitor may be a viable dual-drug strategy for recurrent glioblastoma^[119]^.

Raub *et al*.^[120]^ used *in vivo* glioblastoma models to assess the anti-tumour activity of abemaciclib, while also comparing it to palbociclib. They showed that abemaciclib has anti-tumour activity in intracranial glioblastoma xenograft models, as survival times of tumour-bearing rats increased upon abemaciclib administration^[120]^. More importantly, additive or greater than additive effects were noted when abemaciclib was combined with TMZ^[120]^. In the same study, they showed that abemaciclib crosses the BBB more readily when compared to palbociclib^[120]^, giving hope for its future exploitation in clinical settings for glioma treatment.

Further adding to abemaciclib’s potential as a future therapy for brain tumour patients is its ability to cross the BBB, as demonstrated in a study examining its benefit as a treatment for patients with brain metastases (BM) secondary to breast cancer^[121]^. A clinical trial testing abemaciclib in patients with BM is now closed and results are anticipated (NCT02308020).

TG02 is a brain-penetrating multi-CDK inhibitor^[122]^ [Figure 3]. A recent study showed that TG02 decreased cell viability by targeting CDK9 in patient-derived GBM cells and inhibited tumour growth in an intracranial GBM mouse model^[122]^. TG02 also inhibited cell proliferation and induced cell death in a CDK9 expression-dependent manner in panel of GBM cell lines^[123]^. Additionally, synergism was observed when TG02 was combined with TMZ in cell lines and syngeneic mouse orthotopic GBM model^[123]^. Another group also showed that TG02 reduced cell viability in a panel of GBM cell lines^[124]^, independent of MGMT promoter methylation status. These findings resulted in an on-going clinical trial examining the effectiveness of TG02 in combination with temozolomide in patients with recurrent anaplastic astrocytoma and glioblastoma (NCT02942264). Even though final results are not published, promising results from the Phase I trial have recently been reported, showing that TG02 administration at the maximal tolerated dose and in combination with TMZ has a tolerable toxicity profile^[125]^. A second clinical trial using TG02 in combination with either RT or TMZ, in newly diagnosed elderly glioblastoma or anaplastic astrocytoma patients, is also on-going (NCT03224104). Final outcomes of both trials are eagerly awaited.

The on-going or completed clinical trials using CKIs either as a single treatment or in combination with other drugs or radiotherapy are given in Table 3.

**Table 3:** CKIs used in glioma clinical studies

CKI drugs	Targets	Clinical trial phase	Glioma grade	Combination with other drugs/radiotherapy	ClinicalTrials.gov identifier
Abemaciclib (LY2835219, Verzenio)	CDK 4, 6	Phase II	Recurrent glioblastoma	No	NCT02981940
		Phase II	Glioblastoma	Temozolomide	NCT02977780
		Phase II	Recurrent glioblastoma	Bevacizumab	NCT04074785
		Phase II	Recurrent glioblastoma	No	NCT03220646
		Phase I	Recurrent brain tumour	No	NCT02644460
		Phase II	Oligodendroglioma	No	NCT03969706
Ribociclib (Kisqali)	CDK 4, 6	Phase I	Recurrent glioblastoma/anaplastic glioma	No	NCT02345824
		Phase I	Glioma/Meningioma	No	NCT02933736
		Phase I	Paediatric gliomas/HGG	Everolimus/Radiotherapy	NCT03355794
		Phase I	Recurrent brain tumours	Gemcitabine, sonidegib, trametinib	NCT03434262
Palbociclib (PD-0332991)	CDK 4, 6	Phase II	Oligodendroglioma/Oligoastrocytoma	No	NCT02530320
		Phase I	Central nervous system tumours	No	NCT02255461
		Phase II	Recurrent glioblastoma/gliosarcoma/anaplastic astrocytoma	No	NCT01227434
		Phase I/Phase II	Glioblastoma	Radiotherapy	NCT03158389
		Phase II	Glioma	No	NCT02465060
		Phase II	Recurrent childhood Medulloblastoma/Malignant glioma	No	NCT03155620
		Phase II	Recurrent malignant glioma/recurrent medulloblastoma	No	NCT03526250
		Phase I	Medulloblastoma	Temozolomide, irinotecan	NCT03709680
TG02 (SB1317)	CDK 1, 2, 5, 7, 9	Phase I/Phase II	Recurrent anaplastic astrocytoma/glioblastoma	Temozolomide	NCT02942264
		Phase I	Astrocytoma, Grade III glioblastoma	Temozolomide, radiotherapy	NCT03224104

HGG: high-grade glioma; CKIs: cyclin-dependent kinase inhibitors; CDK: cyclin-dependent kinase

A schematic figure summarising CKIs used in preclinical and clinical studies in gliomas is shown in Figure 3.

## Conclusion

The location of brain tumours and their protection by the BBB means that brain tumours remain the most challenging malignancies to treat. Amongst these, GBM is the most deadly. New treatment approaches are needed that cross the BBB, specifically target the tumour cells and are relatively non-toxic to normal brain cells. CKIs display such attributes and their potential as a novel treatment approach for GBM patients has been supported by multiple preclinical studies. However, similar strong preclinical evidence supported the potential of CDK4/6 inhibition in breast cancer treatment for many years, yet specific and efficient CKIs of CDK4/6 only became recently available. As results from on-going clinical trials emerge and further preclinical studies are conducted, it is hoped that the potential of CDK inhibitors in the treatment of brain tumours, especially GBM, will soon be realised for the benefit of all brain tumour patients.

## Declarations

### Copyright

© The Author(s) 2020.
